# Analysis of Correlation between the Seven Important *Helicobacter pylori* (*H*. *pylori*) Virulence Factors and Drug Resistance in Patients with Gastritis

**DOI:** 10.1155/2020/3956838

**Published:** 2020-08-29

**Authors:** Sinem Oktem-Okullu, Zehra Cekic-Kipritci, Elif Kilic, Nogayhan Seymen, Nesteren Mansur-Ozen, Ugur Sezerman, Yesim Gurol

**Affiliations:** ^1^Department of Medical Microbiology, Acibadem Mehmet Ali Aydinlar University, School of Medicine, Atasehir, Istanbul, Turkey; ^2^Yeditepe University Hospital, Istanbul, Turkey; ^3^Department of Medical Biotechnology, Acibadem Mehmet Ali Aydinlar University, Institute of Health and Science, Atasehir, Istanbul, Turkey; ^4^Department of Biostatistics and Bioinformatics, Acibadem Mehmet Ali Aydinlar University, Institute of Health and Science, Atasehir, Istanbul, Turkey; ^5^Department of Biostatistics and Medical Informatics, Acibadem Mehmet Ali Aydinlar University, School of Medicine, Atasehir, Istanbul, Turkey

## Abstract

The aim of this study is to evaluate the association between seven important *H*. *pylori* virulence factors and antibiotic resistance in patients with gastritis. *H*. *pylori* strains isolated from 33 patients with gastritis were examined. Antimicrobial susceptibilities were tested by GenoType® HelicoDR (Hain Life Science, Germany) test kit and RT-PCR. The virulence-factors were determined using conventional PCR. 39% of patients were resistant for clarithromycin and 27% of patients were resistant for fluoroquinolone. 15% of patients were resistant to both clarithromycin and fluoroquinolone. The *H*. *pylori vacA m1/s2* genotype was the most frequent allelic combination. Patients were possessed the *vacA s1*, *m1* (6.1%); *s1*, *m2* (6.1%); *s2*, *m1* (15.1%); and *s2*, *m2* (3.0%) genotypes. 94% of patients with gastritis were positive for *H*. *pylori napA* gene. Also, there were no *dupA* gene-positive gastritis patients. There was no significant correlation between the *vacA*, *cagA*, *oipA*, *hpaA*, *babA*, *napA*, *dupA*, *ureA*, *ureB* virulence genes, clarithromycin, and fluoroquinolone resistance. Herein, we report that the relationship between the *H*. *pylori napA* gene and gastritis. Although we found a correlation between *H*. *pylori* virulence factor and clinical outcome, there is a need for further studies to enlighten the relation between *H*. *pylori* virulence genes and antibiotic resistance.

## 1. Introduction

*Helicobacter pylori* (*H*. *pylori*) is one of the most important human pathogenic microorganisms with its spiral shape that can endure asymptomatic or can cause several gastrointestinal diseases, ranging in acute and chronic gastritis, gastric and duodenal ulcers, gastric adenocarcinoma, and MALT (mucosa-associated lymphoid tissue) lymphoma [1]. The prevalence rate of *H*. *pylori* differs between the developed countries that have low prevalence and developing countries that have a high prevalence. *H*. *pylori* infection is usually gained during childhood and if it is not treated, it will continue to lifespan [2].

For the treatment of *H*. *pylori*, multiple antibiotic regimens have been evaluated including triple therapy, sequential therapy, quadruple therapy, and levofloxacin-based triple therapy. Antimicrobial susceptibility testing is the best approach to choosing the treatment therapy method. Besides this eradication rates, previous antibiotic exposure and regional antibiotic-resistance patterns should be considered for successful treatment.

In the first-line treatment, a triple therapy regimen is applied including usually a proton pump inhibitor (PPI) combined with clarithromycin and amoxicillin or metronidazole as an antibiotic [3]. Clarithromycin-based therapies were considered to be the best tolerated and safest therapies; However, the efficacy of these triple regimens has decreased due to bacterial resistance to antibiotics [4]. To overcome antimicrobial resistance seen in *H*. *pylori* treatment, new antibiotics and different drug combinations have been evaluated, using fluoroquinolones, rifabutin, furazolidone, and azithromycin 14-day levofloxacin or 5-day levofloxacin concomitant therapy could be an alternative fluoroquinolone therapy regimes where fluoroquinolone resistance is rare [5].

As a highly heterogeneous bacterium virulence of *H*. *pylori* varies geographically. Due to the research in the literature, it is clearly explained that *H*. *pylori* virulence factors have a very important effect on both bacterial pathogenicity and treatment outcome [6].

Vacuolating cytotoxin gene A (*vacA*), cytotoxin-associated gene A (*cagA*), outer inflammatory protein A (*oipA*), the blood group antigen-binding adhesin gene A (*babA*), neutrophil-activating protein A (*napA*), the putative neuraminyllactose-binding hemagglutinin homolog (*hpaA*), duodenal ulcer promoting gene A (*dupA*), urease A (*ureA*), and urease B (*ureB*) are the most important virulence genes of this bacterium that take the role in the invasion, adhesion, and colonization of *H*. *pylori* strains in gastric epithelial cells [7, 8].

The present study is aimed at assessing the relationship between clarithromycin and fluoroquinolone resistance with the seven important *H*. *pylori* virulence factor status.

## 2. Material and Methods

### 2.1. Ethical Approval

A written informed consent about the participation in this study was provided to each patient included in this study. The ethical committee of Yeditepe University approved this study.

### 2.2. Patient Selection

33 patients were included in the study who underwent endoscopy because of gastroduodenal diseases at the Gastroenterology Department of Yeditepe University, in Istanbul, Turkey. They were selected due to the who fulfilled the obtaining expulsion criteria.

### 2.3. Gastric Biopsy Specimens

Gastric biopsy specimens of the patients were taken from the antrum part of the stomach. Freshly taken biopsy specimens were placed into the Nucliswab® Solution (Salubris, USA) and kept at +4°C for overnight, then put at –80°C deep freezer until DNA isolation.

### 2.4. DNA Isolation

To study virulence factors of *H*. *pylori*, DNA was extracted by using QIAamp DNA mini DNA isolation kit (Qiagen, Hilden, Germany)) following the manufacturer's description. All DNA samples were quantified using NanoDrop (ND-2000, THERMO) and maintained in –80°C deep freezer.

### 2.5. Antimicrobial Susceptibility Testing

Fluoroquinolone and clarithromycin resistance were tested by using GenoType® HelicoDR(Hain Life Science, Germany) test kit as described by the following the manufacturer's description. The point mutations related to the clarithromycin resistance was detected by using a real-time PCR- (RT-PCR-) based hybridization assay. *H*. *pylori* 23SrRNA gene-specific primers with probes shown in Table 1 were used, and melting curve analysis was done. For the RT-PCR experiments and hybridization reactions, LightCycler thermocycler (Roche Diagnostics, Neuilly Sur Seine, France) was used.

Real-time PCR reactions were done by using glass capillary tubes with 20 *μ*l volume. 3 *μ*l template DNA, 0.4 *μ*l forward and reverse primers (20 *μ*M each), 0.2 *μ*l sensor and anchor probes (20 *μ*M each), and 2 *μ*l of FastStart DNA Master Hybridization Probes (Roche Diagnostics) were included into each tube. The RT-PCR reaction conditions were 95°C for 10 min as an initial denaturation, 95°C for 0 s for 50 amplification cycles, 60°C for 10 s as annealing, and 72°C for 17 s as an extension. Also, a melting curve step was added to the reaction composed of 95°C for 0 s, 45°C for 30 s to cool (temperature transition rate of 20°C/s), and in the final step, a slow increase in the temperature to 85°C at a rate of 0.1°C/s with the continuous acquisition of fluorescence decrease [11].

### 2.6. Virulence Factor Testing

Extracted patients' DNA samples were used as a template DNA in PCR to detect virulence factors of *H*. *pylori* which are *vacA*, *cagA*, *oipA*, *hpaA*, *babA*, *napA*, *dupA*, *ureA*, and *ureB* genes. The primer sequences and the size of PCR products used in this study were summarized in Table 2. Genomic DNA of *H*. *pylori* isolated from the *H*. *pylori* G27 strain was used as a positive control DNA for PCR assay. Negative control was used by replacing templated DNA with distilled water. Each PCR amplification was carried out in a volume of 15 *μ*l containing 3 *μ*l of 5X Dye Hot Start Master Mix (GeneMark Bio, TAİWAN), 10 *μ*M of forward and reverse primers, and 3 *μ*l of templated DNA. The remaining volume was adjusted with distilled water Different amplification mixtures were prepared using primer pairs belonging to each virulence factor. Each PCR set was included in the negative and positive controls. Conventional PCR reaction conditions were initial denaturation 3 minutes at 95°C, 40 cycles of denaturation 30 seconds at 95°C, annealing 45 second at 60°C and extension 1 minute at 72°C, and final extension 5 minutes at 72°C. Only for *oipA* gene, annealing temperature was used as 59.2°C instead of 60°C. PCR amplifications were performed in a thermal cycler (Bio-Rad T100).

7 *μ*l of PCR products was loaded to agarose gel electrophoresis in 1% agarose gel in 1X Tris Acetic Acid Edta (TAE) buffer at 100 V for 60 min and stained with ethidium bromide. Then, the agarose gels were visualized by the gel imaging system (ChemiDoc™ MP imaging system, Bio-Rad).

### 2.7. Statistical and Bioinformatic Analyses

Our data consists of the presence of virulence factors (cagA, ureA, ureB, napA, hpaA, oipA, and dupA) and drug resistances (clarithromycin resistance and fluoroquinolone resistance), all binary values depicting absence/presence, WT/MUT, and resistant/susceptible, respectively. There is a total of 33 H. *pylori*-positive samples with a mean age of 37.3. Since none of the patients have dupA, that virulence factor was excluded from the study as there will be no meaningful comparison.

For the creation of the correlation tables, we have used Pearson's correlation and performed all analysis in Python, scipy v1.0.0. To observe meaningful differences in outcomes, we performed two-proportion *z*-test. Two-proportion *z*-test was the test of choice here as the sample size is quite small and this test better reflects proportional differences in different sample groups. An alpha value less than or equal to 0.05 was considered significant for both the Pearson and two-proportion *z*-tests.

Our initial tests showed no significant difference in the occurrence of the *H*. *pylori* virulence factors between clarithromycin and fluoroquinolone susceptible/resistance samples. We, therefore, tried a combinatorial approach where we performed the same tests with coupled virulence factors. If both given virulence factors were not present in a patient—for example, both VacA m1 and OipA are absent—then, the new combined feature VacA m1/OipA will also be absent. However, if any or both are present in that patient, then the new feature will be present. In addition to expanding our search space, this also enabled us to check whether these virulence factors occur in tandem or not.

After two proportions of *z*-tests, we have observed a significant difference in combined *VacA m1* and *VacA s2* (*p* value = 0.03) between patients susceptible/resistant to clarithromycin. While VacA_m1/VacA_s2 is present in 55% (11/20) of susceptible patients, it is present in only 23.1% (3/13) of resistant patients.

## 3. Results

### 3.1. Antibiotic Resistance

Due to the antibiotic resistance test results, 39% of patients were resistant for clarithromycin and 27% of patients were resistant for fluoroquinolone. 15% of patients were resistant to both clarithromycin and fluoroquinolone. Resistance rates were shown in Table 3.

### 3.2. Association between Virulence Genotypes and Gastritis

According to the conventional PCR results which were performed using 33 patients with gastritis, virulence gene distributions are shown in Table 4. *H. pylori ureA* and *ureB* gene amplification were found in 73% and 24% of all isolates, respectively. *H*. *pylori vacA s2/m1* (*15.1*%) genotypes were detected as the most frequent allelic combination of the vacA gene among gastritis patients followed by *vacA s1*, *m1* (6.1%); *s1*, *m2* (6.1%); and *s2*, *m2* (3.0%) genotypes. 94% of patients with gastritis were positive for *H*. *pylori napA* gene. Additionally, no gastritis patients were positive for *dupA* gene.

### 3.3. Correlation between Antibiotic Resistance and Virulence Factor Genotype

The relation between the clarithromycin and fluoroquinolone resistance with *H*. *pylori* virulence genes was evaluated (Tables 5 and 6). Due to our data results, there was no significant correlation between the *vacA*, *cagA*, *oipA*, *hpaA*, *babA*, *napA*, *dupA*, *ureA*, *ureB* virulence genes, and clarithromycin, and fluoroquinolone resistance. However, our data showed that there was a significant relationship between vac A s1/m2 allelic variation and clarithromycin susceptibility (*p* value < 0.05).

## 4. Discussion

*Helicobacter pylori* is a common pathogenic bacteria colonizer of the human stomach, and the bacteria have an essential role in the development of different gastroduodenal diseases including gastritis, peptic ulcers, and gastric cancer. Approximately, half of the world's population has been presumed to be infected with this pathogenic bacterium, but only a small amount of infected people develop gastroduodenal diseases. *H*. *pylori* is a highly heterogeneous bacterium, and bacterial virulence genes play a pivotal role in determining the clinical outcome of *H*. *pylori* infection. For the treatment of *H*. *pylori* infection, different types of antibiotic therapy are applied to patients. However, similar to other bacterial infections, drug resistance is the most important cause of eradication failure for this bacterium. Different factors could affect *H*. *pylori* drug resistance. Age and gender, socioeconomic status, host disease status, and virulence factors of the pathogens are the most important factors that influence *H*. *pylori* drug resistance [16].

Clarithromycin and fluoroquinolones are important drugs for the treatment of *H*. *pylori* infection and resistance to these drugs has been associated with eradication failure. In this present study, it was aimed at evaluating the association between seven important *H*. *pylori* virulence genes, *vacA*, *cagA*, *oipA*, *hpaA*, *babA*, *napA*, *dupA*, *ureA*, and *ureB*, with the clarithromycin and fluoroquinolone resistance in *H*. *pylori*-infected patients with gastritis. Due to the PCR results *H. pylori ureA* and *ureB* virulence gene amplification were found in 73% and 24% of all isolates. The analysis results of *H*. *pylori* virulence genes revealed a specific association between the *H*. *pylori napA* virulence gene and *H*. *pylori*-related gastritis. It was found that 94% of patients with gastritis were positive for the *H*. *pylori napA* gene that is one of the most important virulence factors and taking a role in bacterial colonization and gastric inflammation. *H*. *pylori napA* virulence gene behaves as an immune modulator, and it can induce the production of oxygen radicals by the neutrophils [17]. Also, this bacterial virulence factor stimulates neutrophils, monocytes, and dendritic cells to cause induction of interleukin-12 (IL-12), IL-23, and IL-8 expressions [18, 19].

To the best of our knowledge, no report has shown that the direct association of the *H*. *pylori napA* gene with *H*. *pylori*-induced gastritis. *H*. *pylori napA* virulence gene could serve as a biomarker for the development of diagnostic kits to foresee the evolution of *H*. *pylori*-associated gastritis.

*H*. *pylori vacA* and *cagA* virulence genes are the most important virulence-associated genes that play an important role in the pathogenesis of *H*. *pylori*-related gastrointestinal disease. These two genes have different allelic variations, and these variations affect the disease progression. Most of the *H*. *pylori* strains isolated from the patients' biopsy samples have the *vacA* gene. However, the distinction at the signal (s) and mid (m) regions of this gene are responsible for different levels of its cytotoxicity. In this present study, *vacA s2/m1* allelic genotype was the most frequently detected allelic combination of the *vacA* gene detected among gastritis patients. 55% of clarithromycin susceptible gastritis patients had this allelic variation. In the literature, it was shown that s2-type VacA proteins lack detectable vacuolating activity, and our results support this information [20]. In the previous studies, a high level of genetic heterogeneity has been found among *H*. *pylori* strains isolated from various patients, and it is well known that genetic diversity in *H*. *pylori* is geographically and ethnically structured [21].

In the present study, it was also observed in the results that no gastritis patients were positive for the *dupA* gene. Duodenal ulcer promoting (*dupA*) gene is one of the *H*. *pylori* virulence factors that are highly associated with duodenal ulcer development and reduced risk of gastric cancer. Due to our results, *dupA* virulence gene could be used as a specific biomarker for the detection of peptic ulcer diseases [22].

In the literature, some articles show the association between virulence factors and antibiotic resistance in *H*. *pylori* with controversial results. In one of the studies, the absence of *cagA* was correlated with metronidazole resistance [23] and in other studies *vacA* S1 and *cagE* are associated with clarithromycin and metronidazole resistance [24]. However, in some articles, it was found that neither *vacA* nor *cagA* correlates with resistance. In our present study, no statistically significant association was found between the *vacA*, *cagA*, *oipA*, *babA*, *napA*, *hpaA*, *dupA*, *ureA*, and *ureB* virulence genes of *H*. *pylori* with the clarithromycin and fluoroquinolone resistance in *H*. *pylori*-infected patients with gastritis. A major limitation of our work was a relatively low number of patients with specific clinical manifestations. Studies to be carried out by increasing the number and variety of patients may give more accurate results for the virulence factor and drug resistance relation and *H*. *pylori* virulence factors could be used to predict the clinical outcome as well as to determine antibiotic susceptibility.

In conclusion, our current data showed that the analysis results of virulence genes expose a specific association between *H*. *pylori napA* virulence gene and clinical outcome; furthermore, no significant association could be detected among virulence genes and resistance or susceptibility. Also, the *dupA* gene was not found in any patient with gastritis that is thought to be one of the major markers for ulcer diseases. Our data results for the significant correlation between the *vacA s1/m2* and clarithromycin supports the literature information for the lack of vacuolating ability of *vacA s2* variation. Despite advances in our understanding of the correlation between *H*. *pylori* virulence factor and clinical outcome with antibiotic resistance, it is necessary to carry out further investigations to learn more information about the relation between the *H*. *pylori* virulence genes and the antibiotic resistance and clarify the roles of *H*. *pylori* virulence factors.

## Figures and Tables

**Table 1:** Real-time PCR primers and probe sequence.

Virulence factor	Primer name	Primer sequences (5′–3′)	Product size (bp)	Reference
23SrRNA	HPYS/F	TATGGTACCCGCATGATATTCCCATTAGCAGT	267	[9, 10]
	HPYA/R	TAAGAGCTCAGGTTAAGAGGATGCGTCAGTC		[9, 10]
	Sensor probe 5′ labeled with LC-red 640	GGCAAGACGGAAAGACC	2504 to 2520	[9, 10]
	Anchor probe 3′ labeled with fluorescein	TGTAGTGGAGGTGAAAATTCCTCCTACCC	2473 to 2501	[9, 10]

**Table 2:** *H*. *pylori* virulence factor primer sequences used in the study.

Virulence factor	Primer name	Primer sequences (5′–3′)	Product size (bp)	Reference
*ureA*	ure A/F	TGATGGGACCAACTCGTAACCGT	244	[12]
	ure A/R	CGCAATGTCTAAGCGTTTGCCGAA		
*ureB*	ure B/F	AGTAGCCCGGTGAACACAACATCCT	645	[12]
	ure B/R	ATGCCTTTGTCATAAGCCGCTTGG		
*napA*	nap A/F	GAATGTGAAAGGCACCGATT	304	[12]
	nap A/R	ATCGTCCGCATAAGTTACGG		
*babA*	bab A/F	AATCCAAAAAGGAGAAAAAGTATGAAA	832/601	[12]
	bab A/R	TGTTAGTGATTTCGGTGTAGGACA		
*dupA*	dup A/F	TGAGCGTGGTAGCTCTTGAC	584	[12]
	dup A/R	GAGCGCGTTAGCGATATAGG		
*hpaA*	hpa A/F	TAGTGGGATGCAGCCCGCATATTA	534	[12]
	hpa A/R	CGCTATGGCTTGAATGGGTGGTTT		
*oipA*	oip A/F	CATTAAGCGGTGGTTTTGTG	1093	This study
	oip A/R	AGCCAACTAAAGAGCGGTAA		
*cagA*	cagA/F	GTTGATAACGCTGTCGCTTC	350	[13]
	cagA/R	GGGTTGTATGATATTTTCCATAA		
*VacA s1/s2*	VAI/F	ATGGAAATACAACAAACACAC	259/286	[14]
	VAI/R	CTGCTTGAATGCGCCAAAC		
*VacA m1/m2*	VAG/F	CAATCTGTCCAATCAAGCGAG	567/642	[14]
	VAG/R	GCGTCAAAATAATTCCAAGG		
16S rRNA	rRNA/F	TAAGAGATCAGCCTATGTCC	534	[15]
	rRNA/R	TCCCACGCTTTAAGCGCAAT		

**Table 3:** Antibiotic resistance rates of patients with gastritis.

Drug resistance	Patient number and percentage, *n* (%)
Clarithromycin resistance	13 (39)
Fluoroquinolone resistance	9 (27)
Clarithromycin and fluoroquinolone resistance	5 (15)

**Table 4:** Correlation between presence or absence of *H*. *pylori* virulence genes and gastritis.

Virulence genes	Status	Gastritis, *n* (%)
*cagA*	Absent	22 (67)
	Present	11 (33)
*vacA m1*	Absent	24 (73)
	Present	9 (27)
*vacA m2*	Absent	30 (91)
	Present	3 (9)
*vacA s1*	Absent	30 (91)
	Present	3 (9)
*vacA s2*	Absent	23 (70)
	Present	10 (30)
*ureA*	Absent	9 (27)
	Present	24 (73)
*ureB*	Absent	25 (76)
	Present	8 (24)
*napA*	Absent	2 (6)
	Present	31 (94)
*dupA*	Absent	33 (100)
	Present	0 (0)
*oipA*	Absent	32 (97)
	Present	1 (3)
*hpaA*	Absent	22 (67)
	Present	11 (33)

**Table 5:** Association between *H*. *pylori* clarithromycin resistance and virulence genes.

Virulence genes	Status	Susceptible, *n* (%)	Resistant, *n* (%)	*p* value
*oipA*	Absent	20 (100)	12 (92.31)	0.9
	Present	0 (0)	1 (7.69)	
*hpaA*	Absent	14 (70)	8 (61.54)	0.69
	Present	6 (30)	5 (38.46)	
*napA*	Absent	1 (5)	1 (7.69)	0.38
	Present	19 (95)	12 (92.31)	
*ureB*	Absent	15 (75)	10 (76.92)	0.45
	Present	5 (25)	3 (23.08)	
*ureA*	Absent	5 (25)	4 (30.77)	0.36
	Present	15 (75)	9 (69.23)	
*cagA*	Absent	13 (65)	9 (69.23)	0.4
	Present	7 (35)	4 (30.77)	
*vacA s1/s2*	Absent	11 (55)	9 (69.23)	0.21
	Present	9 (45)	4 (30.77)	
*vacA s2*	Absent	13 (65)	10 (76.92)	0.23
	Present	7 (35)	3 (23.08)	
*vacA s1*	Absent	18 (90)	12 (92.31)	0.41
	Present	2 (10)	1 (7.69)	
*vacA m1/m2*	Absent	13 (65)	9 (69.23)	0.4
	Present	7 (35)	4 (30.77)	
*vacA m2*	Absent	18 (90)	12 (92.31)	0.41
	Present	2 (10)	1 (7.69)	
*vacA m1*	Absent	14 (70)	10 (76.92)	0.33
	Present	6 (30)	3 (23.08)	
*VacA_m1_VacA_s2*	Absent	9 (45)	10 (76.92)	0.03^∗^
	Present	11 (55)	3 (23.08)	

**Table 6:** Association between *H*. *pylori* fluoroquinolone resistance and virulence genes.

Virulence genes	Status	Susceptible, *n* (%)	Resistant, *n* (%)	*p* value
*oipA*	Absent	23 (95.83)	9 (100)	0.27
	Present	1 (4.17)	0 (0)	
*hpaA*	Absent	15 (62.5)	7 (77.78)	0.2
	Present	9 (37.5)	2 (22.22)	
*napA*	Absent	2 (8.33)	0 (0)	0,81
	Present	22 (91.67)	9 (100)	
*ureB*	Absent	18 (75)	7 (77.78)	0.43
	Present	6 (25)	2 (22.22)	
*ureA*	Absent	7 (29.17)	2 (22.22)	0.66
	Present	17 (70.83)	7 (77.78)	
*cagA*	Absent	15 (62.5)	7 (77.78)	0.2
	Present	9 (37.5)	2 (22.22)	
*vacA s1/s2*	Absent	15 (62.5)	5 (55.56)	0.64
	Present	9 (37.5)	4 (44.44)	
*vacA s2*	Absent	17 (70.83)	6 (66.67)	0.59
	Present	7 (29.17)	3 (33.33)	
*vacA s1*	Absent	22 (91.67)	8 (88.89)	0.6
	Present	2 (8.33)	1 (11.11)	
*vacA m1/m2*	Absent	16 (66.67)	6 (66.67)	0.5
	Present	8 (33.33)	3 (33.33)	
*vacA m2*	Absent	23 (95.83)	7 (77.78)	0.95
	Present	1 (4.17)	2 (22.22)	
*vacA m1*	Absent	17 (70.83)	7 (77.78)	0,34
	Present	7 (29.17)	2 (22.22)	
*VacA_m1_VacA_s2*	Absent	14 (58.33)	5 (55.56)	0.56
	Present	10 (41.67)	4 (44.44)	

## Data Availability

The data in the article can be used with reference.
